# Tacrolimus versus mycophenolate for AutoImmune hepatitis patients with incompLete response On first-line therapy (TAILOR study): a study protocol for a phase III, open-label, multicentre, randomised controlled trial

**DOI:** 10.1186/s13063-023-07832-w

**Published:** 2024-01-17

**Authors:** Anna E. C. Stoelinga, Maarten E. Tushuizen, Wilbert B. van den Hout, Mar D. M. Rodriguez Girondo, Elsemieke S. de Vries, Amar D. Levens, Dirk-Jan A. R. Moes, Tom J. G. Gevers, Suzanne van der Meer, Hans T. Brouwer, Hendrik J. M. de Jonge, Ynte S. de Boer, Ulrich H. W. Beuers, Adriaan J. van der Meer, Aad P. van den Berg, Maureen M. J. Guichelaar, Joost P. H. Drenth, Bart van Hoek, on behalf of the Dutch Autoimmune Hepatitis Group, Sjoerd F. Bakker, JM Vrolijk, Patrick van der Veek, Nicole F. van Gerven

**Affiliations:** 1 Department of Gastroenterology and Hepatology, Leiden University Medical Center, Leiden, The Netherlands; 2 Department of Biomedical Data Sciences, Leiden University Medical Center, Leiden, The Netherlands; 3 Department of Gastroenterology and Hepatology, Isala Hospital, Zwolle, The Netherlands; 4 Department of Clinical Pharmacy and Toxicology, Leiden University Medical Center, Leiden, The Netherlands; 5 Division of Gastroenterology and Hepatology, Department of Internal Medicine, Maastricht University Medical Center, Maastricht, The Netherlands; 6 European Reference Network RARE-LIVER, Hamburg, Germany; 7 Department of Gastroenterology and Hepatology, University Medical Center Utrecht, Utrecht, The Netherlands; 8 Department of Gastroenterology and Hepatology, Reinier de Graaf Gasthuis, Delft, The Netherlands; 9 Department of Gastroenterology and Hepatology, Jeroen Bosch Hospital, ‘S-Hertogenbosch, The Netherlands; 10 Department of Gastroenterology and Hepatology, Amsterdam University Medical Centre, Location VU University Medical Center, Amsterdam, The Netherlands; 11 Department of Gastroenterology and Hepatology, Amsterdam University Medical Centre, Location Academic Medical Center, Amsterdam, The Netherlands; 12 Department of Gastroenterology and Hepatology, Erasmus University Medical Center, Rotterdam, The Netherlands; 13 Department of Gastroenterology and Hepatology, University Medical Center Groningen, Groningen, The Netherlands; 14 Department of Gastroenterology and Hepatology, Medisch Spectrum Twente, Enschede, The Netherlands; 15 Department of Gastroenterology and Hepatology, Radboud University Medical Center, Nijmegen, The Netherlands; 16 Department of Gastroenterology and Hepatology, Elisabeth Tweesteden Hospital, Tilburg, The Netherlands; 17 Department of Gastroenterology and Hepatology, Rijnstate Hospital, Arnhem, The Netherlands; 18 Department of Gastroenterology and Hepatology, Haaglanden Medical Center, The Hague, The Netherlands; 19 Department of Gastroenterology and Hepatology, Rode Kruis Hospital, Beverwijk, The Netherlands

**Keywords:** AIH, Autoimmune hepatitis, Autoimmune liver disease, Tacrolimus, Mycophenolate mofetil, Randomised controlled trials, Second-line treatment, Complete biochemical response

## Abstract

**Background:**

Autoimmune hepatitis (AIH) is a rare, chronic inflammatory disease of the liver. The treatment goal is reaching complete biochemical response (CR), defined as the normalisation of aspartate and alanine aminotransferases and immunoglobulin gamma. Ongoing AIH activity can lead to fibrosis and (decompensated) cirrhosis. Incomplete biochemical response is the most important risk factor for liver transplantation or liver-related mortality. First-line treatment consists of a combination of azathioprine and prednisolone. If CR is not reached, tacrolimus (TAC) or mycophenolate mofetil (MMF) can be used as second-line therapy. Both products are registered for the prevention of graft rejection in solid organ transplant recipients. The aim of this study is to compare the effectiveness and safety of TAC and MMF as second-line treatment for AIH.

**Methods:**

The TAILOR study is a phase IIIB, multicentre, open-label, parallel-group, randomised (1:1) controlled trial performed in large teaching and university hospitals in the Netherlands. We will enrol 86 patients with AIH who have not reached CR after at least 6 months of treatment with first-line therapy. Patients are randomised to TAC (0.07 mg/kg/day initially and adjusted by trough levels) or MMF (max 2000 mg/day), stratified by the presence of cirrhosis at inclusion. The primary endpoint is the difference in the proportion of patients reaching CR after 12 months. Secondary endpoints include the difference in the proportion of patients reaching CR after 6 months, adverse effects, difference in fibrogenesis, quality of life and cost-effectiveness.

**Discussion:**

This is the first randomised controlled trial comparing two second-line therapies for AIH. Currently, second-line treatment is based on retrospective cohort studies. The rarity of AIH is the main issue in clinical research for alternative treatment options. The results of this trial can be implemented in existing international clinical guidelines.

**Trial registration:**

ClinicalTrials.gov NCT05221411. Retrospectively registered on 3 February 2022; EudraCT number 2021–003420-33. Prospectively registered on 16 June 2021.

## Administrative information

Note: the numbers in curly brackets in this protocol refer to SPIRIT checklist item numbers. The order of the items has been modified to group similar items (see http://www.equator-network.org/reporting-guidelines/spirit-2013-statement-defining-standard-protocol-items-for-clinical-trials/).

**Table:** 

Title {1}	Tacrolimus versus mycophenolate for AutoImmune hepatitis patients with incompLete response On first line therapy: a Randomised trial (TAILOR study)
Trial registration {2a and 2b}.	EudraCT 2021–003420-33 ClinicalTrials.gov Identifier: NCT05221411 EU-CT number: 2023-509491-42-00
Protocol version {3}	Version 2.9, 28-09-2021.
Funding {4}	Grant from ZonMw, number: 10140022010001 And funding from Chiesi Pharmaceuticals b.v. (project number: PA 2019–71111/vjanssen)
Author details {5a}	1. Department of gastroenterology and hepatology, Leiden University Medical Center, Leiden, The Netherlands 2. Department of Biomedical Data Sciences, Leiden University Medical Center, Leiden, The Netherlands 3. Department of gastroenterology and hepatology, Isala Hospital, Zwolle, The Netherlands 4. Department of Clinical Pharmacy and Toxicology, Leiden University Medical Center, Leiden, The Netherlands 5. Division of Gastroenterology and Hepatology, Department of Internal Medicine, Maastricht University Medical Center, Maastricht, the Netherlands 6. Department of gastroenterology and hepatology, University Medical Center Utrecht, The Netherlands 7. Department of gastroenterology and hepatology, Reinier de Graaf Gasthuis, The Netherlands 8. Department of Gastroenterology and Hepatology, Jeroen Bosch Hospital, ‘s Hertogenbosch, The Netherlands 9. Department of Gastroenterology and Hepatology, Amsterdam University Medical Centre, location VU University Medical Center, Amsterdam, The Netherlands 10. Department of Gastroenterology and Hepatology, Amsterdam University Medical Centre, location Academic Medical Center, Amsterdam, The Netherlands 11. Department of Gastroenterology and Hepatology, Erasmus University Medical Center, Rotterdam, The Netherlands 12. Department of Gastroenterology and Hepatology, University Medical Center Groningen, Groningen, The Netherlands 13. Department of Gastroenterology and Hepatology, Medisch Spectrum Twente, Enschede, The Netherlands 14. Department of Gastroenterology and Hepatology, Radboud University Medical Center, Nijmegen, The Netherlands 15. Department of Gastroenterology and Hepatology, Elisabeth Tweesteden Hospital, Tilburg, the Netherlands 16. Department of Gastroenterology and Hepatology, Rijnstate Hospital, Arnhem, the Netherlands 17. Department of Gastroenterology and Hepatology, Haaglanden Medical Center, The Hague, The Netherlands 18. Department of Gastroenterology and Hepatology, Rode Kruis Hospital, Beverwijk, the Netherlands 19. European Reference Network RARE-LIVER
Name and contact information for the trial sponsor {5b}	Prof. dr. B. van Hoek Leiden University Medical Center Department of Gastroenterology and Hepatology, C4P P.O. Box 9600 2300 RC Leiden, the Netherlands b.v_hoek@lumc.nl Phone: 071–5291111/5299756/5263507
Role of sponsor {5c}	The funders have no role in the study design, collection, or management of data. Neither ZonMw nor Chiesi Pharmaceuticals b.v. have access to coded of decoded data. The funders do not play a role in the analysis or publication process of this study.

## Introduction

### Background and rationale {6a}

Autoimmune hepatitis (AIH) is a rare, chronic inflammatory disease of the liver. It is characterised by the presence of circulating autoantibodies, elevated immunoglobulin gamma (IgG), elevated aspartate and alanine aminotransferases (AST and ALT respectively) and interface hepatitis in histology. The simplified or original diagnostic criteria for AIH are used for the diagnosis [1, 2]. Clinical manifestation is very heterogeneous, differing from asymptomatic raised liver enzymes and nonspecific symptoms as fatigue and arthralgia to acute (on chronic) liver failure [3].

The goal of treatment is achieving and maintaining clinical remission. Remission of disease is important to prevent further progression of disease to fibrosis and eventually cirrhosis, decompensated cirrhosis, liver transplantation or liver-related mortality [4–10]. Complete biochemical response (CR) is defined as serum ALT, AST and IgG below the upper limit of normal (ULN) [11, 12].

Standard treatment consists of prednisolone (0.5–1 mg/kg/day) and azathioprine (1–2 mg/kg/day) according to the European guidelines [11]. In case of an adequate response to therapy, steroids can be tapered gradually. In 80–90% of the patients on first-line treatment, adequate biochemical response is seen with a prompt decrease of aminotransferases [11], although around 50% have not achieved CR after a year.

Treatment of patients with an incomplete response or intolerance to prednisone (or budesonide) and azathioprine (or 6-mercaptopurin or 6-thioguanine) remains a clinical challenge. As incomplete response occurs in 20–50% of the patients (50% 1 year after treatment, 20% at 3 years) [13, 14], second-line treatment options are needed. The scientific evidence for second-line therapy currently consists of expert opinions, case series and retrospective studies. In this study, we aim to prospectively compare the biochemical response rate of tacrolimus (TAC) versus mycophenolate mofetil (MMF), both combined with glucocorticoids, as second-line therapy in AIH without CR on first-line therapy.

## Objectives {7}

The primary objective of this study is to investigate the difference in the proportion of patients in complete biochemical response (CR) after 12 months of treatment with TAC compared to MMF, both with glucocorticoids, in patients with AIH and an incomplete response to at least 6 months of first-line treatment.

CR is defined as serum ALT, AST and IgG below the upper limit of normal, conforming to European Association of the Study of the Liver (EASL) guidelines [11].

## Trial design {8}

**Fig. 1:**
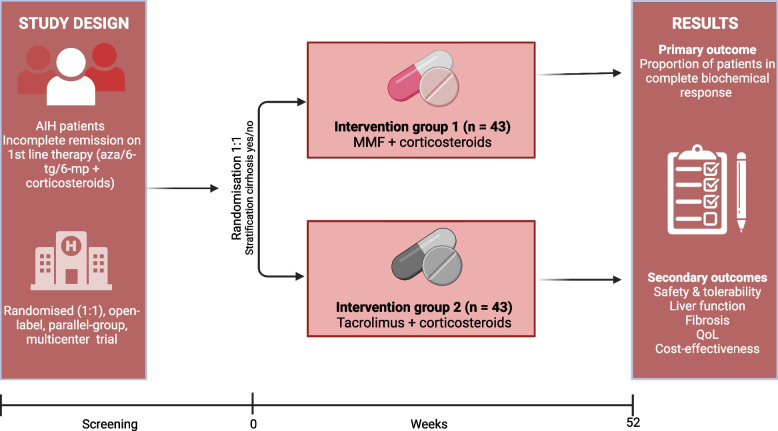
Flowchart of TAILOR study. AIH, autoimmune hepatitis; aza, azathioprine; MMF, mycophenolate mofetil; QoL, quality of life; 6-tg, thioguanine; 6-mp, mercaptopurine

This is a study protocol for a phase III, multicentre, open-label (i.e. non-blinded) randomised, controlled superiority trial, with parallel group assignment and 1:1 allocation. The protocol is written in accordance with the Standard Protocol Items: Recommendation for Interventional Trials (SPIRIT) guidelines [15]. Figure 1 shows a schematic diagram of the trial as the SPIRIT figure.

## Methods: participants, interventions and outcomes

### Study setting {9}

This multicentre study will be conducted at the gastroenterology and hepatology outpatient clinics of seven academic centres and three large teaching hospitals in the Netherlands. Other Dutch hospitals can recruit patients and refer them to participating hospitals for screening and inclusion. A list of participating centre can be found at ClinicalTrials.gov under NCT05221411.

### Eligibility criteria {10}

#### Inclusion criteria

- Patient is older than 18 years old.
- Probable or definite auto-immune hepatitis according to the original or simplified IAIHG criteria (> 10 points pre-treatment on the original criteria or > 6 points on the simplified criteria) [1, 2].
- Incomplete responder on at least a half year of first-line treatment, including azathioprine/6-MP/6-TG and prednisolone or budesonide.
- ALT 1.5–10 × ULN for at least 2 months.
- Patient is capable of understanding the purpose and risks of the study, has been fully informed and has given written informed consent to participate in the study.

#### Exclusion criteria

- Presence of decompensated liver disease, defined as ascites, coagulopathy (INR > 1.5), encephalopathy, variceal bleed, hepato-pulmonal syndrome, hepatorenal syndrome or HCC in the past 6 months
- Signs of other liver diseases as metabolic-dysfunction associated steatotic liver disease (MASLD), Wilson disease, hemochromatosis, alcoholic liver disease or viral hepatitis B/C/D
- Clinical diagnosis of overlap/variant syndrome with PBC or PSC
- Liver transplantation in the medical history or currently on the waiting list for liver transplantation
- Incompliance with therapy during the last 12 months
- Active infections during inclusion including latent tuberculosis and HIV co-infection
- Allergic or hypersensitive to TAC or MMF
- An estimated glomerular filtration rate (eGFR) of < 60 mL/min
- Pregnancy or intention to become pregnant in the next 12 months
- Use of TAC or MMF in the past
- Malignancy in the medical history in the past 5 years, or current use of chemotherapy.

### Who will take informed consent? {26a}

Patients are recruited at the outpatient clinics of all participating hospitals as well as in recruiting hospitals. They will be asked to participate by their treating physician. The local principal investigator will explain the study to potential study subjects. Subsequently, patients will receive a patient information form (PIF). After 1 week, patients will be contacted by the central study coordinator, to whom questions can be asked and by whom additional clarification can be given. After having given informed consent, a screening visit (Tx) will be planned.

### Additional consent provisions for collection and use of participant data and biological specimens {26b}

Patients will provide informed consent for the collection of back-up samples of serum and plasma, which will be performed in several centres. These samples will be stored locally and will be used for central end-point analyses. Throughout the study, additional research questions may arise. Ancillary studies can be performed on blood samples.

### Interventions

#### Explanation for the choice of comparators {6b}

Several retrospective studies have reported on the use of TAC as a second-line therapy in autoimmune hepatitis. In several case series, TAC was given to patients unresponsive to prednisone and/or AZA. The first study of TAC in AIH was performed by van Thiel et al [16]. They studied the use of TAC for induction treatment of AIH and found a decrease in ALT in 80% of the patients. In several subsequent (relatively) small case series, TAC was given as second-line therapy for AIH, whereas remission rates differed between 20 and 92% [14, 17–22].

The use of MMF for AIH, both in case of intolerance for first-line treatment and in case of insufficient response, has been published by our group [23]. Retrospective cohort studies indicate that in AIH patients with intolerance or insufficient response on first-line therapy, with MMF complete remission can be induced in 0–22% of patients [23–25]. One study reported a remarkable proportion of 57% remission with MMF [26]. There appeared to be a difference in remission rates between patients started on MMF for intolerance to first-line treatment or for insufficient response to first-line treatment [23–25].

Both TAC and MMF are mentioned as possible second-line therapy in guidelines and literature. In one paper, CR on MMF was reported to be 22% in patients with an incomplete response, which is lower when compared to the CR rate in patients with intolerance to AZA. A cohort of treatment naïve AIH patients has also been treated successfully with MMF [27–29].

Because of a more favourable profile of adverse effects, MMF should possibly be favoured as second-line treatment for AIH, and not TAC, although it might be less effective [30]. TAC could be more effective than MMF as second-line therapy for incomplete response. However, the aforementioned studies are all retrospective cohort studies. No randomised trials for second-line therapies for AIH have been performed.

#### Intervention description {11a}

##### Group one: Prolonged-release tacrolimus (Envarsus®)

For this study, TAC will be used, preferably 0.75 mg, 1 mg and 4 mg tablets of Envarsus® from Chiesi Pharmaceuticals. Envarsus is a once-daily prolonged-release formulation with more stable tacrolimus levels [31, 32]. Patients in this arm will start with oral 0.07 mg/kg/day of meltdose tacrolimus once daily. If Envarsus is unavailable, other forms of TAC (e.g., Advagraf, Adport, Dailiport, Prograft) may be used, with the appropriate dosing scheme, since different forms of TAC have different bioavailability. Tacrolimus has a narrow therapeutic window and highly variable pharmacokinetics. Therapeutic drug monitoring (TDM) will be performed to reach a target AUC of 160 µg h/L (20% interval 128–196) tacrolimus. This is compatible with trough levels of 3.6–7.4 µg/l [33–35]. Tacrolimus can have several adverse effects. Due to a narrow therapeutic window, therapeutic drug monitoring is necessary to prevent underexposure, which can result in reduced effect, and overexposure, which can lead to side effects including hypertension, tremor, headache, renal toxicity and hyperkalaemia. Like with any other immunosuppressive drug, there is an increased risk of infections.

##### Group 2: mycophenolate mofetil (Cellcept®)

For this study, 500 mg tablets of CellCept® from F. Hoffmann-La Roche AG Pharmaceuticals will be used. If not available, generic forms of MMF may be used. Patients in the MMF arm will receive a maximum total dose of 2000 mg daily, split-dose. Patients will commence with a dose of 500 mg twice daily. When tolerated, doses will be titrated to 1000 mg twice daily after week 2. The most common adverse effects are gastrointestinal toxicity, leukopenia, thrombopenia and increased risk of infections.

#### Criteria for discontinuing or modifying allocated interventions {11b}

Subjects can leave the study at any point during the follow-up. Investigators can decide to withdraw a subject from the study when:

- A flare of AIH (ALT > 10xULN) occurs
- Other urgent medical reasons (e.g. pregnancy (for patients assigned to MMF) or acute (on chronic) liver failure)

In case of severe adverse effects or eGFR <50 ml/min, the dose of study medication will be halved. If severe side effects persist despite halving the dose, study medication will be discontinued. If the eGFR is < 40ml/min, study medication will also be discontinued. In case study medication is discontinued, all follow-up visits and assessments will still be performed with the exception of the therapeutic drug monitoring.

If loss of remission, defined as ALT >1–3× ULN after reaching CR, occurs or when ALT reaches 3×10× ULN during the study, corticosteroid dose will be increased to the dose at screening, while trial participation can be continued as per discretion of the treating physician.

#### Strategies to improve adherence to interventions {11c}

TAC and MMF will be packed and labelled as study medication according to Good Manufacturing Practice (GMP) guidelines by the Department of Pharmacy of the Leiden University Medical Center (LUMC). Local study investigators will perform drug accountability using a drug accountability log. Patients will be asked to take their medication and empty boxes to the close-out visit to control compliance. Additionally, the *Basel Assessment of Adherence to immunosuppressive medIcations Scale* (BAASIS) will be used to check compliance. The questionnaires will be filled out during each clinic visit or will be sent electronically by e-mail. Reminders will be sent after 14 days.

#### Relevant concomitant care permitted or prohibited during the trial {11d}

New medication may be prescribed at the discretion of the treating physician. No new immunosuppressants may be used, besides the corticosteroids already in use at screening. If a flare of AIH (ALT > 10 × ULN) occurs during the study period, patients will receive the standard of care from the treating physician and trial participation will end. Study subjects are not allowed to participate in other trials investigating pharmaceutical agents for AIH.

#### Provisions for post-trial care {30}

Participants who suffer harm from participating in the study are covered by the liability insurance of the LUMC with a maximum of €650,000 per subject, €5,000,000 per study and €7,500,000 per year. The insurance applies to the damage that becomes apparent during the study or within 4 years after the end of the study.

### Outcomes {12}

#### Primary outcome

Proportion of patients with CR after 12 months of treatment with TAC compared to patients with MMF treatment in AIH patients with incomplete remission to at least 6 months of first-line treatment.

CR is defined as ALT, AST and IgG below the upper limit of normal. The endpoint will be expressed as a hazard ratio with 95% confidence interval.

#### Secondary outcomes

The secondary objectives are:

1. Safety and tolerability—number and severity of side effects; rate of stopping treatment due to side effects; serum creatinine and potassium; blood pressure; blood glucose levels and incidence of new-onset diabetes; number of (opportunistic) infections; tremor; diarrhoea
2. Proportion of patients with CR after 6 months
3. Cumulative corticosteroid dose
4. Change of AST, ALT and IgG at 6 and 12 months versus baseline and between groups at the same time points
5. Liver function: total bilirubin, albumin, INR and MELD-score after 6 and 12 months between groups
6. Fibrosis: liver stiffness as measured by elastography and blood fibrosis markers (Enhanced Liver Fibrosis test)
7. Quality of life (questionnaires) using the validated liver disease symptom index (LDSI), short-form 36 (SF-36) and EQ5D
8. Cost-effectiveness: using modified Productivity Cost Questionnaire (iPCQ) and Medical Consumption Questionnaire (iMCQ) questionnaires

### Participant timeline {13}

A schematic overview of study visits and assessments is shown in Table 1. The total duration of the study will be the undefined period of time between screening and the start of the investigative product and 12 months of follow-up.

**Table 1:**
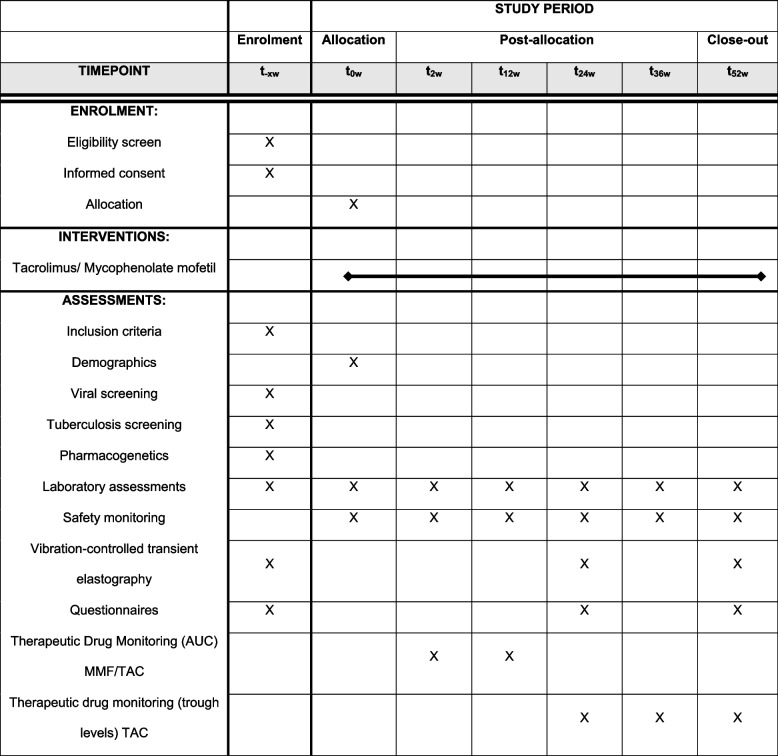
The schedule of enrolment, interventions and assessments in the Standard Protocol Items: Recommendations for Interventional Trials (SPIRIT) figure

* AUC* area under the curve, *MMF* mycophenolate mofetil, *TAC* tacrolimus

### Sample size {14}

A sample size calculation is based on the log-rank test for comparing two groups, a follow-up of 12 months and the assumption of exponential survival times. The expected difference between groups was formulated in terms of relative risk derived from the expected cumulative incidences in the two groups according to previous literature. Papers on MMF for insufficient response in EU and North America reported 13%, 21% and 34% CR respectively, and review of the literature on TAC in adult AIH patients was also taken into account [22, 23, 25, 36]. Assuming proportions of CR at the end of follow-up of 0.22 in the MMF group and 0.53 in the TAC group with a two-sided *α* of 0.05 significance level and power of 1 − *β* = 0.8 to determine the difference in proportions of 0.31, results, in *N* = 38 patients per group. Taking into account a 10% loss to follow-up (e.g. due to side effects), this results in *N* = 43 patients per group. Thus, we aim to include a total of 86 patients.

### Recruitment {15}

Recruitment will take place at regular outpatient clinic visits. Patients will be approached by their treating physician. Study visits will take place during regular outpatient clinic visits, providing a low threshold for participation in follow-up.

### Assignment of interventions: allocation

#### Sequence generation {16a}

Participants will be randomised 1:1, with a variable block randomisation between azathioprine or MMF, stratified for the presence of cirrhosis (yes/no), based on recent vibration-controlled transient elastography (VCTE) (> 16kPa) or liver biopsy (Metavir F4), using the electronic case report form (eCRF) by Castor Electronic Data Management (Castor EDC).

#### Concealment mechanism {16b}

Participants, treating physicians and local and central study coordinators are blinded for the randomisation process, but not for medication use during the study (because of the need for blood level monitoring of TAC) and not for the outcome of individual participants.

#### Implementation {16c}

Randomisation will be done by the central study coordinator. Assessors of the final outcome will be blinded. The allocation sequence is unknown to the central study coordinator.

### Assignment of interventions: blinding

#### Who will be blinded {17a}

The design of this study is open-label. This entails that only the outcome assessors will be blinded during analyses. The assigned study arm will be known for both patients and all research staff (including the primary caregiver and principal investigator).

#### Procedure for unblinding if needed {17b}

As this is an open-label study, unblinding of participants, local and central study coordinators and primary caregiver will not be required.

### Data collection and management

#### Plans for assessment and collection of outcomes {18a}

At the screening visit, data on demographics, medical history and medication use is collected after obtaining informed consent. Simultaneously laboratory assessments are done, including screening for systemic infections and pharmacogenetics, and VCTE is performed. After inclusion, participants visit the outpatient at different timepoints (Table 1). During these visits, laboratory tests are done, TDM is done using the area under the curve measurements (visits T_2_ and T_12_) or trough levels (only for TAC on visits T_24_, T_36_, T_52_), and (serious) adverse events ((S)AEs) are reviewed. During specified study visits, health-related quality of life (HRQoL) questionnaires are administered and VCTE is done.

#### Plans to promote participant retention and complete follow-up {18b}

Participants visit the outpatient clinic at an interval of 3 months, as per the standard of care in most participating centre. Patients who are referred from a recruiting centre can visit the participating centre at a six-monthly interval, having the remaining study visits in the recruiting centre as part of standard care. For patients who withdraw from the study due to (severe) side effects of toxicity, follow-up visits will be performed with the exception of TDM. No additional data of patients discontinuing treatment for other reasons will be collected.

#### Data management {19}

All data will be entered electronically in case report forms in Castor EDC. Data are collected by the site personnel or the coordinating investigator. After signing informed consent, participants will receive a trial identification number.

#### Confidentiality {27}

All data and specimens will be entered and stored in a coded fashion. The code key will be kept at the participating sites. Access will be limited to involved researchers, who are responsible for the processing of the data. The dataset analysed during the current study and statistical code can be requested through the principal investigator of the sponsor site.

#### Plans for collection, laboratory evaluation and storage of biological specimens for genetic or molecular analysis in this trial/future use {33}

At screening, weeks 24 and 52, additional serum samples will be taken for central end-point analyses. All biological specimens stored during the study are stored for possible additional analyses at a later stage.

### Statistical methods

#### Statistical methods for primary and secondary outcomes {20a}

Intention-to-treat analysis will be used throughout the study for primary and secondary endpoints. All randomised patients will be included in the intention-to-treat analysis. CR at 12 months will be reported as percentage. A Kaplan–Meier survival analysis with log-rank test will be performed to assess the rapidity of reaching CR. Time to CR will be censored at 12 months. In the unlikely event that a subject is withdrawn from the study due to liver-related mortality or liver transplantation, a competing risk analysis according to Fine and Gray will be performed.

All parameters will be summarised by the treatment group using descriptive statistics. Mean, standard deviation, median and range will be used for continuous variables. Counts and percentages will be used for categorical variables. For normally distributed continuous variables, Student’s *T*-test will be used, for not normally distributed continuous variables Mann–Whitney *U* test will be used. For categorical variables, the chi-square test or Fisher exact test will be used. For repeated measurements, paired *T*-test and Wilcoxon signed rank test will be used where appropriate. Confounding will be tested where appropriate.

The economic evaluation comparing the MMF and TAC policies will include a trial-based cost-effectiveness analysis (1-year healthcare costs per complete biochemical remission) and a model-based cost-utility analysis (life-long societal costs per QALY, calculated from the EQ-5D-5L).

Statistical analyses will be performed with Statistical Package for Social Sciences (SPSS), version 24.0 or higher. Two-sided tests will be used and* p*-values < 0.05 will be deemed significant.

#### Interim analyses {21b}

No interim analysis is planned in this study. Safety of the interventions will be monitored by registering (serious) adverse events. In accordance with Section 10, subsection 4, of the WMO, the sponsor will suspend the study if there is sufficient ground that continuation of the study will jeopardise the subject health or safety.

#### Methods for additional analyses (e.g. subgroup analyses) {20b}

No subgroup analysis will be performed in this study.

#### Methods in analysis to handle protocol non-adherence and any statistical methods to handle missing data {20c}

In the event of missing data, this will be dealt with accordingly using additional statistical analyses (e.g. imputation). The primary analysis will be an intention-to-treat analysis. Every patient who has received at least one dose of study medication will be included in the ITT analysis.

#### Plans to give access to the full protocol, participant-level data and statistical code {31c}

After publication of the final manuscript, data may be available upon reasonable request. The dataset analysed during the current study and statistical code can then be requested through the principal investigator of the sponsor site, as is the full protocol.

### Oversight and monitoring

#### Composition of the coordinating centre and trial steering committee {5d}

Leiden University Medical Center is the coordinating centre of this study. Both the principal investigator and the coordinating investigator are employed by the coordinating centre. The coordinating investigator is responsible for the day-to-day support of participating and recruiting sites. Additionally, data collection and analyses will be done once the study is finished. The principal investigator is updated on the study weekly. Additionally, the Dutch Autoimmune Hepatitis Working Group (DAIHWG) is updated biannually on the study’s progress during meetings. The DAIHWG consists of hepatologists with an interest for AIH from various (study) sites.

#### Composition of the data monitoring committee, its role and reporting structure {21a}

It was not deemed necessary by the medical ethical committee to constitute an independent data safety management board for this study.

#### Adverse event reporting and harms {22}

AEs are defined as any undesirable experience occurring to a subject during the study, whether or not considered related to the investigational product. All adverse events reported spontaneously by the subject or observed by the investigator or his staff will be recorded. A SAE is any untoward medical occurrence or effect that (a) results in death; (b) is life-threatening (at the time of the event); (c) requires hospitalisation or prolongation of existing inpatients’ hospitalisation; (d) results in persistent or significant disability or incapacity; (e) is a congenital anomaly or birth defect; and (f) requires medical or surgical intervention to preclude of any other important medical event that did not result in any of the outcomes listed above due to medical or surgical intervention but could have based upon appropriate medical judgement. Elective hospital admissions are not considered SAEs. The principal investigator of the participating site will report the SAE to the coordinating investigator within 24 h. The latter will report the medical ethical committee via Toetsingonline.nl within 7 (death) or 15 (life threatening event) days.

#### Frequency and plans for auditing trial conduct {23}

The TAILOR study is monitored at all participating sites by a set monitor assigned by the LUMC. Monitoring will be performed at set intervals determined in the monitoring plan. This interval is initially set once every year, taking into consideration the recruitment status and protocol adherence. The (internal) monitor may decide to intensify the monitoring interval. Auditing upon invitation of the hospital may occur, when deemed necessary. The frequency of auditing is unknown.

#### Plans for communicating important protocol amendments to relevant parties (e.g. trial participants, ethical committees) {25}

Substantial amendments will be submitted to the medical ethical committee of the LUMC before implementation, with the exception of amendments for the immediate safety of participants. Important protocol modifications, influencing patients, will be communicated to the central and local ethics committees as appropriate.

### Dissemination plans {31a}

Collected data will not yet be made available, since recruitment is still ongoing. The results of this trial will be submitted for publication in a scientific journal. In addition, the results will be presented in the ClinicalTrials.gov database. Outcomes of this study will be shared with the scientific community, patient representatives, sponsor and patients through conferences, meetings, newsletters and other relevant media.

## Discussion

There is an unmet clinical need for the evaluation of second-line treatment in AIH. In clinical practice, rates of CR on first-line therapy are moderately low, at least initially [13, 37]. The TAILOR study is a multicentre, phase 3b, randomised, open-label clinical trial to evaluate the efficacy and safety of TAC versus MMF in patients with AIH and no CR. Reaching CR in AIH is of ultimate importance to prevent disease progression to cirrhosis, liver transplantation or liver-related mortality [4–10, 38]. The absence of complete remission 1 year after starting treatment in AIH is related to a lower liver-transplant-free survival [10]. In this trial, we aim to provide an evidence base for an improved organogram-like set-up for treating patients with AIH and incomplete CR.

This study has several potential strengths. At this moment, no registered second-line therapy for AIH exists. To our knowledge, this is the first interventional study comparing two frequently used second-line therapies (albeit off-label) for AIH. Several retrospective studies have demonstrated a beneficial effect of both treatment strategies in patients with AIH and intolerance to azathioprine or for incomplete CR [14, 17–27]. Both drugs are registered and extensively used for the prevention of graft rejection in transplant recipients. Furthermore, using the same glucocorticoid tapering schedule after CR has been reached in both study arms enables better comparison. Additionally, the TAILOR study is carried out by the Dutch Autoimmune Hepatitis Study group that cooperates very closely on both national and international levels (i.e. in the International Autoimmune Hepatitis Group (IAIHG)).

However, some important limitations of this trial must be considered. A multicentric set-up is required to reach complete recruitment. Administrative affairs may cause delays in opening study sites, limiting enrolment. After starting as a national study, an international extension of the study might be preferable. Furthermore, the open-label set-up may introduce some form of recall bias. Additionally, TAC, and especially Envarsus, is not always available at all sites. Consequently, different forms of TAC may be used throughout the study. Additionally, experience with TAC in non-transplant centres is limited. Therefore, it is possible that these study centres may be reluctant to include patients in the study. Active involvement of the coordinating investigator may encourage less experienced study sites to include participants. Lastly, recruiting hospitals are given the opportunity to refer patients to participating sites for study visits only (T0, T24, T52). This study set-up may simplify participation for patients who prefer their own treating physician.

In conclusion, this study should answer the question whether TAC or MMF is the second-line drug of choice, in combination with glucocorticoids, in case of not reaching CR on first-line therapy in AIH. The findings of this study are expected to inform the current clinical practice guidelines on the management of patients with AIH and incomplete CR to standard therapy.

## Trial status

The trial was registered on 16–06-2021 in the European Union Drug Regulating Authorities Clinical Trials Database (#2021–003420) and on ClinicalTrials.gov (#NCT05221411, https://www.clinicaltrials.gov/study/NCT05221411). The first patient was randomised on 24–01-2022. The trial is ongoing and actively recruiting. To date, 5 patients of the 86 patients have been randomised. Expected completion of recruitment is in 2025.

## Data Availability

Patients are coded by a numeric randomisation code. Considering the ongoing nature of the study, collected data is not made publicly available. After publication of the final manuscripts, data may be available upon reasonable request. The dataset analysed during the current study and statistical code can be requested through the principal investigator of the sponsor site, as is the full protocol.

## Abbreviations

AIH: Autoimmune hepatitis
ALT: Alanine aminotransferases
AST: Aspartate aminotransferases
AUC: Area under the curve
AZA: Azathioprine
BAASIS: Basel Assessment of Adherence to immunosuppressive medIcations Scale
CR: Complete biochemical response
EASL: European Association of the Study of the Liver
eCRF: Electronic case report form
EU: European Union
eGFR: Estimated glomerular filtration rate
GMP: Good Manufacturing Practice
HIV: Human immunodeficiency virus
HrQOL: Health-related quality of life
IAIHG: International Auto Immune Hepatitis Working Group
IgG: Immunoglobulin gamma
INR: International normalised ratio
LUMC: Leiden University Medical Center
MELD: Model end-stage liver disease
MMF: Mycophenolate mofetil
NAFLD: Non-alcoholic fatty liver disease
PIF: Patient information form
PBC: Primary biliary cholangitis
PSC: Primary sclerosing cholangitis
(S)AE: (Serious) adverse events
SPIRIT: Standard protocol items: recommendation for interventional trials
TAC: Tacrolimus
TDM: Therapeutic drug monitoring
ULN: Upper limit of normal
VCTE: Vibration-controlled transient elastography
